# Rhizoplane Bacteria and Plant Species Co-determine Phosphorus-Mediated Microbial Legacy Effect

**DOI:** 10.3389/fmicb.2019.02856

**Published:** 2019-12-10

**Authors:** Ming Lang, Shuikuan Bei, Xia Li, Thomas W. Kuyper, Junling Zhang

**Affiliations:** ^1^College of Resources and Environment, Southwest University, Chongqing, China; ^2^Centre for Resources, Environment and Food Security, College of Resources and Environmental Sciences, China Agricultural University, Beijing, China; ^3^Key Laboratory of Plant-Soil Interactions, Ministry of Education, Beijing, China; ^4^School of Life Science, Shanxi Datong University, Datong, China; ^5^Soil Biology Group, Wageningen University, Wageningen, Netherlands

**Keywords:** soil microbiome, high-throughput sequencing, microbial ecological network, rhizocompartment, long-term phosphorus fertilization

## Abstract

Much effort has been directed toward increasing the availability of soil residual phosphorus (P). However, little information is available for the P fertilization-induced biotic P legacy and its mediation of plant P uptake. We collected microbial inocula from a monoculture maize field site with a 10-year P-fertilization history. A greenhouse experiment was conducted to investigate whether bacterial communities, as a result of different P-fertilization history (nil P, 33 and/or 131 kg P kg ha^–1^ yr^–1^), affected the growth of a conspecific (maize) or heterospecific (clover) plant, at two levels of current P application (5 and 30 mg P kg^–1^ soil; P_5_ and P_30_). Deep amplicon sequencing (16S rRNA) was used to determine the maize and clover root-associated bacterial microbiome in different rhizocompartments (rhizoplane, rhizosphere, bulk soil). For both maize and clover, rhizocompartment and host identity were the dominant factors shaping bacterial assemblages, followed by P supply level and the inoculum effect was smallest. Bacterial operational taxonomic unit (OTU) numbers decreased from bulk soil to rhizoplane, whilst specific OTUs were enriched from bulk soil to rhizoplane. A clear hierarchical habitat filtering of bacterial communities was observed in the rhizoplane of the two plant species. The functional prediction of dominant bacterial taxa in the rhizoplane differed between clover and maize, and clover microbiota were more closely associated with P metabolism and maize with carbon cycling. More connected and complex interactions were observed in the clover rhizoplane compared to maize. The microbial legacy effect caused by long-term P fertilization is overridden by host identity and rhizocompartment. Our results highlight the importance of crop diversification in improving P efficiency. The fine-tuning of rhizosphere microbiome in host metabolism indicates that the functions of microbial communities should be integrated into P management to increase P use efficiency and sustainable food production.

## Introduction

Phosphorus (P) is essential for plant growth and development (Raghothama, 1999; Vance et al., 2003), and often is the limiting nutrient in agricultural soils worldwide (Sattari et al., 2012). However, most of the P applied to agroecosystems in the form of mineral fertilizers, manures and waste is retained in the soil as inorganic and organic P that is only sparingly available to plants, due to the high P sorption capacity of most soils which results in low P acquisition and use efficiency by most crops (Condron et al., 2013). Consequently, a substantial fraction of applied P accumulates as residual P in soil. This legacy-P stock in agricultural soils could be sufficient to sustain global crop yields for approximately 100 years without yield decline if it can be made available (Zhu et al., 2018). Much efforts are directed toward increasing the availability of this legacy P to crop plants (Menezes-Blackburn et al., 2018). Soil microorganisms in particular root and rhizosphere microbiota attract much attention, as these microbiomes play key roles in determining plant health and productivity (Berendsen et al., 2012).

Many soil bacteria and fungi have considerable potential to enhance the bioavailability and utilization of residual P sources. Studies have devoted to the impact of P fertilization on the diversity and composition of soil microbial communities, and the understanding of the mechanisms by which microbes mine or scavenge soil P (Richardson and Simpson, 2011). Phosphorus fertilization significantly increased the diversity and abundance of genes involved in P cycling (Su et al., 2015). Microbes involved in P cycling excrete phosphatases (Tabatabai, 1994), to mobilize orthophosphate. Some microbes contain phosphate starvation-inducible (psi) genes and act as part of the phosphate starvation regulon. These microbes have the capacity to synthesize phosphomonoesterases and phosphodiesterase (Vershinina and Znamenskaya, 2002). The *Oxalobacteraceae* (mainly *Massilia* and *Herbaspirillum*), *Klebsiella*, and some species of *Burkholderia* and *Bacillus* were enriched in rock phosphate-amended soil compared to triple superphosphate-treated soil.

There is a compositional transition from bulk soil via rhizosphere to the rhizoplane, with the rhizoplane acting as a regulatory gate for microbial entry into the host (Van der Heijden and Schlaeppi, 2015). A clear hierarchical filtering of microbiota by soybean and alfalfa was reported in different rhizocompartments, e.g., nodule and root endophytes, rhizosphere, and root zone (Xiao et al., 2017). In contrast, bacterial communities in the nodule and roots of *Lotus japonicus* displayed parallel but not consecutive patterns (Zgadzaj et al., 2016). Currently we lack a predictive framework for understanding the biotic and abiotic factors governing the observed rhizosphere microbiome differences. Both environmental filtering and plant traits may co-determine the differentiation of root microbiota. A recent study reported that the structure of root microbiome community was shown to be co-coordinated by the interactions between plant P starvation with plant immune responses (Castrillo et al., 2017). Hence understanding whether and how differences in root and rhizosphere microbiomes respond to P stress gradients are crucial for deciphering the nature of plant-microbial interactions in response to P supply.

Root microbiomes co-evolve with plants. Root microbiota consistently influence plant growth and health (Richardson and Simpson, 2011; Dombrowski et al., 2017; Hartman et al., 2017). In both agro- and natural ecosystems, a preceding plant often leaves a legacy for subsequent-plant growth (Van der Putten et al., 2013). Negative plant–soil feedback (PSF) has been frequently observed in continuous monoculture, driven by the accumulation of specific pathogens. The negative feedback can be eliminated or transformed into positive PSF when plants are rotated with other crops (Ocimati et al., 2017). Simultaneously, under nutrient deficiency conditions, plants may preferentially select root microbiota to assist host nutrient acquisition, also leading to a positive PSF (Revillini et al., 2016). Under sufficient nutrient supply, P-solubilizing bacteria decreased in the rhizosphere (Mander et al., 2012). Culture-dependent studies on interactions between P supply and microbes showed that phosphate mineralization was higher in low P soil than that in high P soil (Mander et al., 2012). These results imply that long-term nutrient amendments may select microbes that have different effects on the fitness of microbes and host plant. We need to understand whether crop-associated microbial communities have shifted as a result of P fertilization, and whether potential beneficial interactions between plants and the associated microbes become weaker over time due to microbial niche diversification.

In this study, we were interested in whether and to what extent fertilizer P-mediated microbial legacy impacted on subsequent con- or hetero-specific plant P uptake. Maize has been monocultured for 10 years, and we chose both maize and clover as the host plants, as nodule-forming legumes tend to behave differently from cereal species due to their symbiotic relationships with diverse bacteria and high P demand for N-fixation from the atmosphere. Cropping designs are generally based on above-ground plant functional-trait complementarity with the ignorance of plant-microbe interactions. Integration of microbial dynamics into cropping systems will ultimately help to directly and indirectly manipulate microbial communities to enhance P use efficiency and sustain the productivity of agricultural crops.

We hypothesized that, (1) compared to soil P legacy, different plant species have stronger effects on selecting microbial communities in the rhizoplane and rhizosphere; (2) rhizosphere microbiota are potentially more related to P mobilization under low P condition, or more related to carbon metabolism under higher P supply; and (3) microbial networks in the rhizoplane are characterized by more functionally interrelated operational taxonomic units (OTUs) than those in the rhizosphere and bulk soil. Network analysis was used to explore the organization and dynamics of microbial niches (Duran-Pinedo et al., 2011; Faust and Raes, 2012).

## Materials and Methods

### Sampling Site and Soil Preparation

Soil from a long-term P fertilization trial at the Shangzhuang Experimental Station of the China Agricultural University, Beijing (39°59′N, 116°17′E) was used in this study. The climate is warm and sub-humid with an average annual temperature of 13.2°C and precipitation ranging from 213 to 840 mm. The soil type is a calcareous alluvial soil with a silt loam texture typical of the region. The trial was established in 2007, consisting of a gradient of P fertilizer application with annual inputs of 0, 11, 22, 33, 44, 66, and 131 kg P ha^–1^ as calcium superphosphate. Seeds of spring maize (cv. Zheng dan 958; grown as monoculture) were annually sown in May.

Soil samples (inoculum) were collected from the field plots. Here, we used I_0_ (nil P), I_33_ (33 kg P ha^–1^), and I_131_ (131 kg P ha^–1^). In total 12 soil inoculum were collected (3 P fertilization levels × 4 replicates). The substrate soil used for the experiment was collected from the top 20 cm of the same experiment but from plots with no maize. The soil was air-dried, sieved (2 mm), and sterilized by γ-radiation (>25 k Gray, Beijing Radiation Application Research Center). The physico-chemical properties of the soil were: organic C content 11.5 g kg^–1^, total N 0.72 g kg^–1^, available N 8.5 mg kg^–1^ (NO_3_^–^ and NH_4_^+^), Olsen-P 3.5 mg kg^–1^, NH_4_OAc-K 32.3 mg kg^–1^, and pH 8.2 (1:5, soil to water). Mineral nutrients were mixed thoroughly into the soil at the following concentrations (mg kg^–1^ dry soil): 100 N as NH_4_NO_3_, 5 and 30 P (P_5_ and P_30_) as KH_2_PO_4_ and 100 K as K_2_SO_4_. The added nutrients were mixed thoroughly with the soil.

### Experimental Design

**FIGURE 1:**
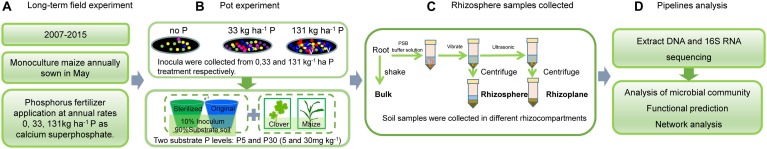
Flow diagram of the experiment.

The pot experiment was conducted in a greenhouse at China Agricultural University. A four-factorial experiment was used, with maize and clover, three soil inocula (I_0_, I_33_, and I_131_), either whole soil or sterilized, and two P levels (P_5_ and P_30_). Each treatment had four replicates resulting in 96 pots. Flow diagram of the key experimental arrangements showed in the Figure 1.

### Harvest and Sampling

Maize was harvested after 65 days. Clover was harvested twice: after 105 days in P_5_ and after 85 days in P_30_. In the P_5_-treatment, the growth of clover was very slow, and only at 105 days they were at the same ontogeny as those in P_30_.

Shoots and roots of maize and clover were separately harvested. Shoot samples were heated at 105°C for 30 min and then oven-dried (72 h, 75°C), weighed, and ground for nutrient analysis. Plant P concentration was determined by the molybdo-vanadophosphate method after samples were digested with concentrated H_2_SO_4_ and H_2_O_2_.

Soils loosely attached to the plant roots were removed by gentle shaking, which was considered as bulk soil. Soils closely associated with plant roots were separated by vigorous vortex (Mobio, 13111-v-220, United States) with 50 ml of sterile phosphate buffered saline (PBS) solution. The roots were stirred vigorously with sterile forceps in order to remove all soil from the root surface. We centrifuged (Sigma-Aldrich, sigma3k15, Germany) that solution and considered the sediment as rhizosphere soil. After removing all the soils, the roots were put into the tube with 15 ml PBS. The roots in the tube were sonicated for 30 s at 50–60 Hz (output frequency 42 kHz, power 90 W, ultrasonic cleaner, Branson Ultrasonics, United States). The roots were removed and discarded, and we centrifuged the solution. This part of the sediment was considered as rhizoplane soil. The efficacy of this procedure for removing microbes from the rhizoplane on whole non-sonicated roots and thrice-sonicated roots was shown by scanning electron microscopy (SEM; Supplementary Figure S1).

### DNA Extraction, 16S rRNA Gene Amplification and MiSeq Sequencing

We extracted total DNA from sampled three rhizocompartments (rhizoplane, rhizosphere and bulk soils) using a fast^®^ DNA SPIN Kit (MP Biomedicals, Cleveland, OH, United States) following the manufacturer’s instructions. We did not sample the endosphere, as the rhizoplane is a strong filter for microbial communities. The quantity and quality of DNA samples were assayed using a NanoDrop ND-1000 UV-visible light spectrophotometer (NanoDrop Technologies, Wilmington, DE, United States). After DNA extraction samples were stored at −20°C for high-throughput sequencing analysis.

Soil DNA was amplified and barcoded with primer set 338F and 806R. The primers 338F (5′-ACTCCTACGGGAGGC AGCAG-3′) and 806R (5′-GGACTACHVGGGTWTCTAAT-3′) target the V4–V5 hypervariable regions of the bacterial 16S ribosomal RNA gene. Each barcode unique sequence was added to the forward primer in each sample. The PCR was carried out with 10 ng template DNA, 0.8 μl of each primer both at 5 μM, 4 μl 5 × FastPfu Buffer, 2 μl 2.5 mM dNTPs, 0.4 μl FastPfu Polymerase (TransGen Biotech, Beijing, China), and made up to a final volume of 20 μl with ddH_2_O, and the thermal conditions were 95°C for 3 min, 27 cycles of 95°C for 30 s, 55°C for 30 s and 72°C for 45 s of extension, followed by 72°C for 10 min. PCR products were then purified and mixed in equimolar ratios to obtain quantitative sample DNA library that was further used for sequencing from the adaptor. Finally, sequencing was conducted using an Illumina MiSeq platform and sequences were submitted to the NCBI database under accession number SUB3124089. The raw sequences were demultiplexed and quality-filtered using the Quantitative Insights Into Microbial Ecology (QIIME) tool kit (version1.19). The primers were removed, and sequences with a quality score of <20, or any truncated reads shorter than 50 bp were eliminated. OTUs were then clustered at the 97% sequence similarity level using the Usearch program that provides clustering, chimera checking, and quality filtering in QIIME. Finally, the most abundant sequence for each OTU was selected as the representative OTU and taxonomic annotations were assigned to each OTU’s representative sequence against the SILVA (SSU117/119) 16S rRNA database for bacteria (Yilmaz et al., 2014).

### Functional Prediction of Soil Bacteria Communities

To better understand the potential functional contributions of the observed shifts in microbial composition in the rhizoplane (the rhizoplane acts as a critical gate for controlling microbial entry into the host tissue), we assigned functional profiles for the two P treatments (P_30_ and P_5_) using the PICRUSt software package^1^ (Langille et al., 2013). This technique estimates genomic copy number of each gene family (KO) in an OTU from an environmental sample based on the position of those OTUs in a reference phylogeny of complete microbial genomes. Functional-profile assignments were made based on partial 16S rRNA gene sequences, and the sequences were mapped to the Greengenes 13_8 reference phylogeny using QIIME (Morrow et al., 2015). Following functional assignment, the obtained profiles of gene family abundances across samples were summarized into KEGG Pathways using PICRUSt. The table containing the functional gene abundances for each sample at KO abundance table and subsystem level 3 of KEGG Orthologs were downloaded for analysis.

### Network Analysis

The relatively high-abundance OTUs (>300 sequences per OUT, a total of 1781 OTUs) were retained for the network analysis. SparCCs (Sparse Correlations for Compositional data) were constructed to infer bacterial OTU–OTU interaction networks as described by Faust et al. (2012), Hu et al. (2019), and Perez-Jaramillo et al. (2019). Generally, the significance of SparCCs was assessed with a permutation test with 999 permutations of random selections in the presence/absence matrices. SparCCs above 0.5 or below −0.5, and statistical significance (*P* < 0.01) were extracted and imported to network analyses (Faust et al., 2012; Hu et al., 2019). The topology of networks was calculated using the Gephi platform. Visualization of the co-occurrence network was also achieved using the Gephi platform (Bastian et al., 2009).

### Statistical Analysis

Significant differences in P concentration and contents of maize and clover among different inoculum with different P levels and sterilization treatments were analyzed using three-way ANOVA followed by the Duncan’s multiple-comparison analysis using SPSS 20.0 (SPSS, Inc., Chicago, IL, United States) software package.

The low-abundance OTUs were eliminated if they did not have a total of at least five reads across all samples. Estimates of α-diversity were based on OTU abundance matrices and included OTU number and Shannon–Wiener diversity calculated by the function diversity in the R package Vegan (Hammer et al., 2001). The bacterial β-diversity was investigated using the analysis of similarities (ANOSIM) and permutational multivariate analysis of variance (PERMANOVA) using the R vegan package (version 3.3.1) with 999 permutations (Anderson, 2001). Principal coordinate analysis (PCoA) was used to compare β-diversity between samples based on the Bray–Curtis distance. This was performed in the R software package (version 3.3.1) using the “ape” library (Paradis et al., 2004). PERMANOVA of β-diversity variance was performed based on Bray–Curtis distance. Ternary plots were drawn in R using the vcd package (Meyer et al., 2015). Constrained analysis of Principal Coordinates (CAPs) was performed to test the amount of variance explained by P level and inoculum in each rhizocompartment of the two plant species, with the capscale function of R package (Oksanen et al., 2013) To assess the responses of predicted microbial function profiles to substrate P supply, we re-organized the data to sum the abundances of each predicted functions at the P_5_ and P_30_ treatments separately. Analysis of Metagenomic Profiles (STAMP) software package was used (Parks and Beiko, 2010) to compare the predicted functional profile of different substrate P supplied for each plant species. *P*-values were calculated using the two-sided Fisher’s exact test, while confidence intervals were calculated using the Newcombe–Wilson method and the correction was made using Benjamini–Hochberg false discovery rate (Benjamini and Hochberg, 1995).

## Results

### Shoot P Content

Available P concentrations in both maize and clover were significantly higher at P_30_ than at P_5_. No significant difference in available P was observed among different inoculum treatments, except that available P in maize at P_5_ when inoculated with I_131_ was significantly higher than when inoculated with I_0_ and I_33_ (Supplementary Table S1). No significant difference in shoot P concentration of both maize and clover was observed among different inocula and between both P levels (except at I_131_). In general, live inoculum significantly increased shoot P content of both maize and clover, except that the significant positive effect was diminished at P_30_ in maize. At both P levels, shoot P content of maize in the I_131_ treatment was significantly higher than that in the I_0_ and I_33_ treatments (Supplementary Table S1).

### Bacterial α-Diversity and Community Structure (β-Diversity)

After quality control, a total of 6,180,927 high-quality sequences were obtained with a median read count per sample of 37,234 (range: 25,345–44,934), with an average of 437 bp read length. The high-quality reads were clustered into 7,132 microbial OTUs using ≥97% sequence identity. Only 30 low-abundance OTUs (<5 total reads) were discarded, resulting in 7102 OTUs for the final analysis.

**FIGURE 2:**
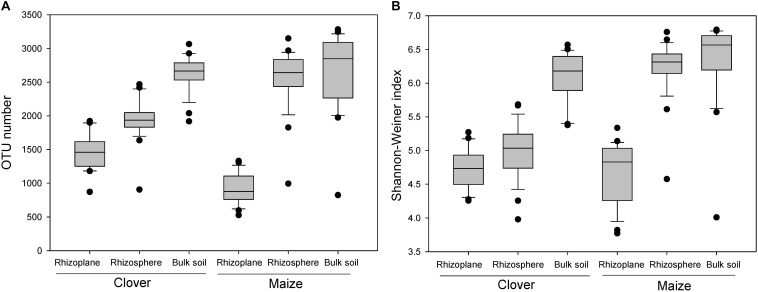
Boxplot of alpha diversity of the bacterial community in different rhizocompartments (rhizoplane, rhizosphere, bulk soil) of maize and clover plants. **(A)** OTU number; **(B)** Shannon–Weiner index.

There was a strong compartment effect on the bacterial α-diversity in the rhizosphere of both maize and clover (*P* < 0.001). OTU numbers and Shannon-Wiener indexes revealed a similar diversity gradient from the rhizoplane to bulk soil (Figures 2A,B), with rhizoplane < rhizosphere ≈ bulk soil in maize, and a significant increase of diversity in the order of rhizoplane < rhizosphere < bulk soil in clover plant (Figures 2A,B).

**FIGURE 3:**
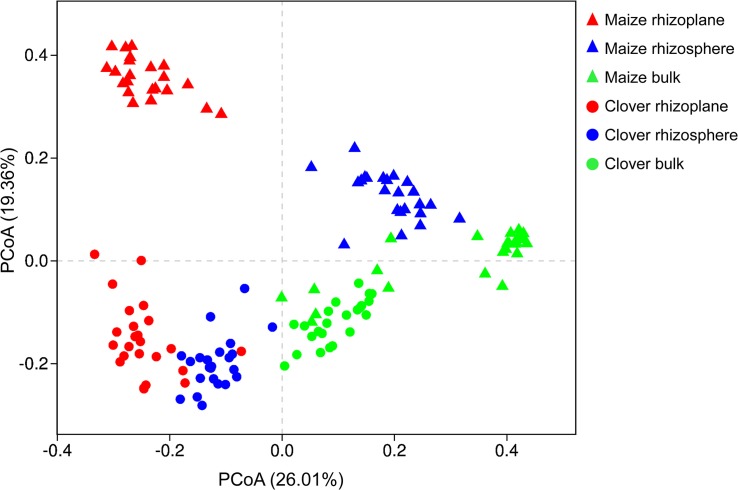
Bacterial communities in different rhizocompartments (rhizoplane, rhizosphere, and bulk) of clover and maize plants. Principal coordinate analysis (PCoAs) plots of the OTU-based Bray–Curtis distance between all samples. The variance explained by each PC axis is given in parentheses.

**FIGURE 4:**
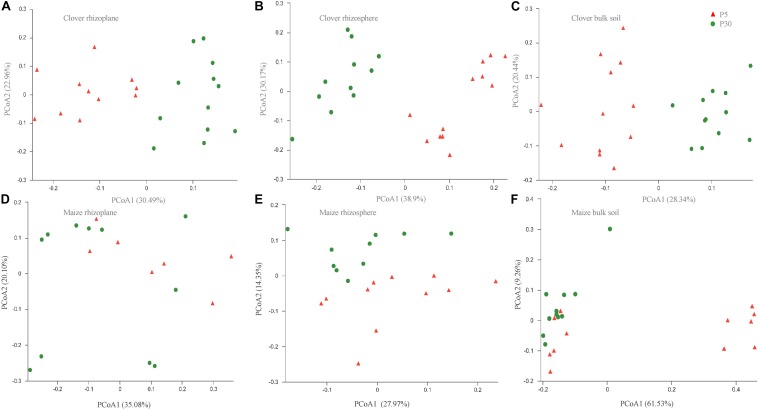
Bacterial communities in different rhizocompartments (rhizoplane, rhizosphere, and bulk) of clover and maize plants in response to substrate P levels. **(A)** Clover rhizoplane; **(B)** clover rhizosphere; **(C)** clover bulk; **(D)** maize rhizoplane; **(E)** maize rhizosphere; and **(F)** maize bulk. Principal coordinate analysis (PCoAs) plots of the OTU-based Bray–Curtis distance between all samples. The variance explained by each PC axis is given in parentheses. P5 and P30 represented the substrate P fertilizers with 5 and 30 mg P kg^– 1^ soil.

Principal coordinate analysis of all samples based on Bray–Curtis dissimilarities revealed that the first two axes explained 45.4% of the variance, with the first axis (26.0% variance explained) separating samples by the compartment and the second axis (19.4%) by plant species. In both maize and clover, there was a clear differentiation among different rhizocompartments (Figure 3), and this was further supported by the PERMANOVA analysis based on a Bray–Curtis distance metric (maize: *R*^2^ = 0.440, *P* < 0.001; clover: *R*^2^ = 0.306, *P* < 0.001) (Supplementary Table S2). Compared to rhizocompartment, substrate P supply level (maize: *R*^2^ = 0.021, *P* = 0.009; clover: *R*^2^ = 0.025, *P* = 0.007) and inoculum (maize: *R*^2^ = 0.031, *P* = 0.010; clover: *R*^2^ = 0.027, *P* = 0.077) had much weaker effects on bacterial communities (Figure 3 and Supplementary Table S2). Bacterial community structure in different rhizocompartment as affected by P level showed that P supply in the recruitment of rhizosphere microbiota was stronger for clover than for maize (Figure 4 and Supplementary Table S3). Constrained analysis of principal coordinates (CAPs) on Bray–Curtis distance was further performed to quantify the variance attributable to each experimental variable. When conditioned on the rhizocompartment, bacterial communities varied significantly between P_5_ and P_30_ (Supplementary Table S4), and the difference was more distinct for clover than for maize. Furthermore, bacterial communities based on the Bray–Curtis distance metric between the original and sterilized inoculum treatments were significantly different (maize: *R*^2^ = 0.212, *P* = 0.001; clover: *R*^2^ = 0.142, *P* = 0.003; Supplementary Figure S2 and Supplementary Table S5).

### Taxonomic Characterization of Bacterial Communities in Different Rhizocompartments

The rhizocompartments followed a clear hierarchical filtering. We observed highly specialized rhizoplane bacterial communities characterized by lower numbers of OTUs occurring with high relative abundance.

*Proteobacteria* were significantly enriched in the rhizoplane of both plants (maize: 72.3%; clover: 61.2%) and rhizosphere (maize: 56.1%; clover: 48.9%). *Bacteroidetes* were also enriched in the rhizosphere. By contrast, other phyla, including *Actinobacteria*, *Acidobacteria*, *Chloroflexi*, *Gemmatimonadetes* were depleted in the rhizosphere and rhizoplane (Supplementary Figure S3).

**FIGURE 5:**
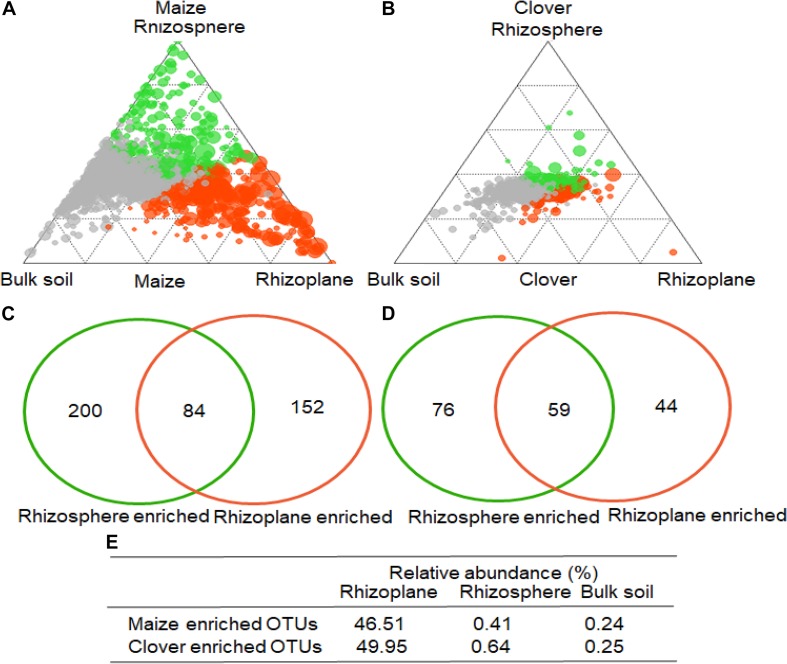
Operational taxonomic unit enrichment in the rhizoplane of maize and clover roots. The ternary plot depicts the rhizocompartments (rhizoplane, rhizosphere, and bulk soil). Relative abundance of all OTUs (>0.05%) in at least one sample in **(A)** maize or **(B)** clover (*n* = 72). Each point corresponds to an OTU. The size of each circle represents its relative abundance, and the position of each circle is the contribution of OTUs in the indicated compartment to the total relative abundance. Orange and green circles marked OTUs enrich in rhizoplane, rhizosphere, whereas gray circles represent OTUs that are not significantly enriched. A Venn diagram comparing differentially enriched OTUs in the rhizosphere relative to bulk soil, and rhizoplane relative to rhizosphere in **(C)** maize and **(D)** clover plants. The number in the overlapping part of the circles depicts the consecutive OTUs from rhizosphere to rhizoplane. **(E)** The relative abundances of the consecutive enriched OTUs account for rhizoplane, rhizosphere, and bulk soil, respectively.

To identify indicator OTUs in the rhizoplane and rhizosphere, we employed linear model analysis to determine the enriched bacterial OTUs (Figures 5A,B). In maize, 284 and 236 OTUs were significantly enriched in the rhizoplane and rhizosphere, compared to bulk soil (Figure 5C), while 84 OTUs were almost exclusively confined to the rhizoplane, with their relative abundances accounting for 46.5, 0.4, and 0.2% in the rhizoplane, rhizosphere and bulk soil (Figure 5E). Compared to bulk soil, 135 and 103 OTUs were significantly enriched in the rhizoplane and rhizosphere of clover (Figure 5D). 59 OTUs were almost exclusively confined to the rhizoplane, with their relative abundances accounting for 50.0, 0.6, and 0.3% in the rhizoplane, rhizosphere and bulk soil (Figure 5E).

Taxonomic analysis showed that the enriched OTUs in the rhizoplane mainly belonged to *Proteobacteria*, with relative abundances of 48.9 and 28.6% in the rhizoplane of clover and maize (Supplementary Figure S4A). Other enriched OTUs belonging to *Actinobacteria*, *Bacteroidetes*, *Chloroflexi*, and *Verrucomicrobia*, were also highly enriched in maize but less in clover (Supplementary Figure S4A). At class level, the α-, β-, and γ-*Proteobacteria* were the dominant taxa, and the relative abundances of α- and γ-*Proteobacteria* were significantly higher in clover than in maize. The relative abundances of *Actinobacteria*, *Cytophagia*, and *Verrucomicrobia* were significantly lower in maize than those in clover. OTUs affiliated with *Sphingobacteria*, *Chloroflexi*, and *Thermomicrobia* were unique for maize rhizoplane (Supplementary Figure S4B). At the order level, *Pseudomonadales*, *Corynebacteriales*, *Micrococcales*, *Rickettsiales*, and *Sphingobacteriales* were uniquely found in the rhizoplane of maize (Supplementary Figure S4C).

### Functional Profiling of the Rhizoplane Microbiota

**FIGURE 6:**
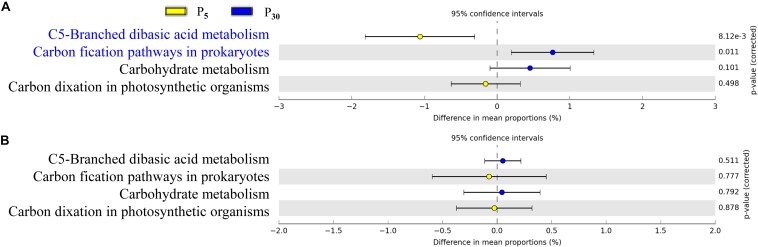
Statistical comparison (Student’s *t*-test) between the predicted functions in rhizoplane bacterial OTUs of **(A)** maize and **(B)** clover grown at P_5_ vs. P_30_. The OTUs relevant to carbon metabolism were based on the KEGG level 3 database using the Benjamini–Hochberg *P*-value correction (*P* < 0.05).

**FIGURE 7:**
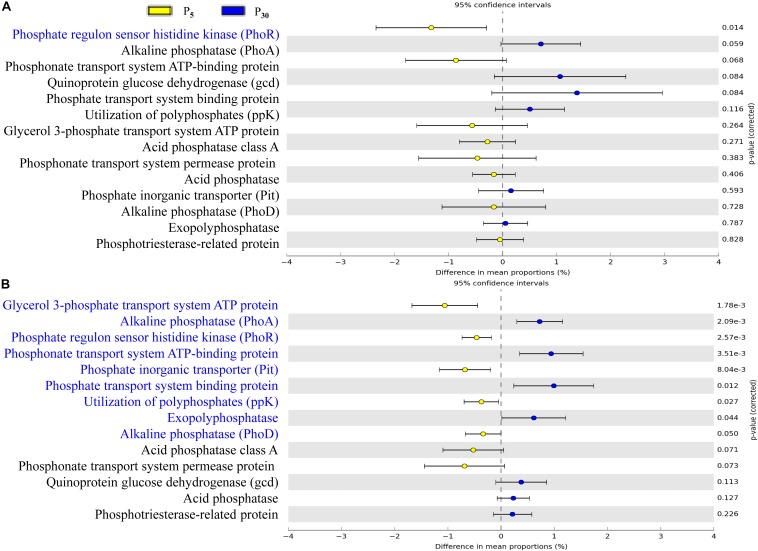
Statistical comparison (Student’s *t*-test) between the predicted functions in **(A)** maize and **(B)** clover grown at P_5_ vs. P_30_ in rhizoplane bacterial OTUs relevant to P metabolism based on the KO abundance database using the Benjamini–Hochberg *P*-value correction (*P* < 0.05).

To gain further insights into the potential functioning of rhizoplane microbiomes, the functional genes from the KEGG pathway sequence hierarchy level 3 (including four genes relevant to carbon metabolism (Figures 6A,B) and KEGG orthology (KO) abundance were evaluated including 14 genes relevant to P metabolism (Figures 7A,B). The predicted functional profiles in response to P supply for maize and clover differed remarkably. The pathways associated with carbon metabolism and carbon fixation were significantly different between P_30_ and P_5_, but the effect was significant only in maize (Figures 6A,B). The genes such as glycerol 3-phosphate transport system ATP protein, phosphate inorganic transporter (Pit), utilization of polyphosphates (ppk) and exopolyphosphatase were higher at P_5_ than those at P_30_. By contrast, the abundances of functional genes with respect to P metabolism and cycling were significantly different in clover (Figure 7B) but not in maize plants (Figure 7A). The genes of phosphate regulon sensor histidine kinase (Pho R), alkaline phosphatase (Pho A), phosphonate transport system ATP-binding protein and phosphate transport system binding protein were higher at P_30_ than at P_5_ (Figure 7A). In maize, no significant difference was observed between the two P levels, except that phosphate regulon sensor histidine kinase (Pho R) was higher at P_30_ than that at P_5_ (Figure 7B).

### Microbial Networks in Rhizoplane Compartments

The results from co-occurrence network showed that the community structure of bacteria in both plant species differed remarkably among the rhizoplane, rhizosphere, and bulk soil. The average clustering coefficients for the rhizosplane and rhizosphere of maize plants were lower than that for bulk soil. Whilst they were opposite for clover plants (Supplementary Table S6). Compared to that of maize, the network of clover showed longer average path length, higher modularity, and average connectivity in the rhizoplane at both P supply levels (Supplementary Table S6). In addition, the network patterns, e.g., the average modularity, connectivity and complexity in rhizoplane, rhizosphere, and bulk soil were generally higher at P5 relative to at P30 (Supplementary Table S6). The top10 bacteria belonged to different taxa in different rhizocompartments and at substrate P levels (Supplementary Table S7). The potential core microbiome for maize mainly included *Xanthomonadaceae*, *Methylobacteriaceae*, *Roseiflexaceae*, and *Gemmatimonadaceae*, and *Chitinophagaceae*, *Coxiellaceae*, and *Comamonadaceae* were for clover.

## Discussion

In the present study, the inoculation of field soils significantly affected bacterial communities (Supplementary Figure S3 and Supplementary Table S5). The recovery of the bacteria from field soils of different P fertilization history exerts strong effect on bacterial communities. Remarkable difference in rhizosphere microbiome was observed between clover and maize (Figure 3), which confirms previous studies on microbial communities in the rhizosphere of different crops. Legume and grass differentially select microbial communities (Zhou et al., 2017). Long-term (12 years) cover cropping with four leguminous species significantly enhanced soil microbes that were related with N and C metabolism (Dinesh et al., 2006). The difference between clover and maize partly could be associated with root exudates and P demand by different plant species. We did not measure root exudation in our study. However, previous studies showed that both exudate rates and composition differed markedly between legume and maize. Maize root exudates, such as sugars, organic acids, aromatics, and enzymes influence nutrient availability, pH, as well as soil microbiome (Peiffer et al., 2013). Rhizosphere phosphatase activity in legume is higher than that of grass (Li et al., 2004). The nature of N_2_-fixation and high demand for P during N_2_-fixation may also drive the diversification of rhizosphere microbiome in clover. We found that *Proteobacteria* constituted the dominant phylum that was enriched on the rhizoplane of both plant species (Supplementary Figure S4). This is consistent with previous studies on legume (Li et al., 2014; Hartman et al., 2017; Yang et al., 2017) and maize (Peiffer et al., 2013; Niu et al., 2017). *Proteobacteria* are involved in the degradation of complex organic compounds (Revillini et al., 2016; Wei and Jousset, 2017). In addition, *Verrucomicrobia*, *Actinobacteria*, and *Chloroflexi* were much more abundant in the rhizoplane of clover compared to maize (Supplementary Figure S4). *Verrucomicrobia* as an indicator of soil fertility generally play roles in degrading contaminants (Cardman et al., 2014). *Actinobacteria* play diverse roles in their association with host plants (Barka et al., 2016). *Rhizobiales* fix atmospheric N_2_ and are important for plant N nutrition. The differentiation between clover and maize was further shown at class and order levels (Supplementary Figure S4).

Our results indicated that rhizocompartment was the main factor affecting bacterial community structure (Figures 2, 3 and Supplementary Table S2). Previous studies on the maize microbiome showed remarkable differences between bacterial communities in the rhizosphere or rhizoplane vs. bulk soil (Silva et al., 2017). The decrease of α-diversity with root proximity was consistent with other crops including wheat (Fang et al., 2017), soybean (Xiao et al., 2017), alfalfa (Xiao et al., 2017), and maize (Niu et al., 2017). In both clover and maize, the ordination of OTUs revealed a clear separation between bulk soil, rhizosphere and rhizoplane samples, with a smaller role for P levels and inoculum. Specific OTUs were consecutively enriched from bulk soil to rhizoplane (Figures 4A,B), and the relative abundances of consecutive OTUs accounted for nearly 50% for both clover and maize on the rhizoplane (Figures 4C–E). The strong specificity in the bacterial communities in the rhizocompartment compared to bulk soil has been consistently shown in other plant species (Reinhold-Hurek et al., 2015; Van der Heijden et al., 2016; Beckers et al., 2017). Rhizocompartment followed a clear hierarchical filtering of microbiota, and such filtering has been previously reported for two legume species (Xiao et al., 2017), *Acacia* and *Phaseolus vulgaris* (Miranda-Sánchez et al., 2016), rice (Edwards et al., 2015) and *Arabidopsis thaliana* (Lundberg et al., 2012). Filtering in different rhizocompartments was mainly due to niche characteristics (Xiao et al., 2017). The rhizosphere and root zone microbial communities were largely influenced by soil type, and the nodule and root endophytes were primarily determined by plant species. Microbial communities in both bulk soil and rhizosphere of soybean showed that rhizosphere bacterial community was selected based on functional cores related to the metabolisms of nitrogen, iron, P and potassium (Mendes et al., 2014). In the present study, we did in-depth analyses on the microbial network (Supplementary Figure S5 and Supplementary Tables S6, S7). Rhizosphere networks consisted of few related, highly abundant and connected species, although how plant traits link with belowground network is still missing (De Vries and Wallenstein, 2017). In the present study, compare to that of maize, the network of clover plants had longer average path length, higher modularity and average connectivity in the rhizoplane (Supplementary Table S6), This indicates that the bacterial communities of clover are highly connected, and organized in the manner of highly cooperate and complexity. Wei et al. (2019) also showed that the co-occurrence network of healthy plants had longer average path length and higher modularity compared to that of the diseased plants. Legume plants require high P to sustain N_2_-fixation, and whether this is closely related to the bacterial assembly deserves further investigations. Furthermore, P supply level significantly affected microbial interactions and network patterns, as shown by the increased average modularity, connectivity and complexity for all compartments at P5 relative to at P30 (Supplementary Table S6). Similarly, P availability is the major variable modulating rhizosphere microbial community of peanut in acidic soils (Chen et al., 2018). Nutrient additions often altered the soil environment by changing pH or availability of carbon or P, which in turn altered the ecological networks (Barberan et al., 2012).

The top 10 bacteria taxa in the rhizoplane based on degree identification were important in the network. The core microbiome in rhizoplane network of both plant species was unique. The core microbiome for maize mainly included *Xanthomonadaceae*, *Methylobacteriaceae*, *Roseiflexaceae*, and *Gemmatimonadaceae*, and *Chitinophagaceae, Coxiellaceae*, and *Comamonadaceae* were for clover (Supplementary Table S7). Previous studies showed that *Xanthomonadaceae*, *Methylobacteriaceae*, and *Gemmatimonadaceae* exhibited the capability of solubilizing P, indicating that maize may recruit phosphorus solubilizing bacteria to assist P acquisition in the rhizoplane (Mander et al., 2012; Chen et al., 2018). The genus of *Lysobacter* in the maize rhizoplane belonged to *Xanthomonadaceae*, which is a rich source for the production of novel antibiotics, such as β-lactams containing substituted side chains, macrocyclic lactams (Hashizume et al., 2004). The presence of *Lysobacter* in the maize rhizoplane may be associated with pathogen defense because the inoculum was from the long-term monoculture maize fields. *Chitinophagaceae* could break the stable C-P coupling metabolism to promote C and P cycling (Seweryn et al., 2015), which may be beneficial for P uptake by clover plants.

P availability (P_5_ vs. P_30_) modified bacterial communities in both plant species (Figure 4 and Supplementary Table S3), indicating resource supply is also affecting the bacterial assemblage in the rhizocompartment. Similarly, P fertilization was the main factor for bacteria communities in intercropped durum wheat and faba bean (Tang et al., 2016). Both the form and amount of mineral fertilizer affected the diversity, structure and functioning of soil microbial communities in the field (Van der Bom et al., 2018). In our study, the importance of P supply in the recruitment of rhizosphere microbiota was stronger for clover than for maize (Supplementary Table S3), as the growth of clover was greatly inhibited in the sterilized I_0_ treatment. The alteration of rhizosphere microbiota tends to result in potential functional differentiation in terms of P acquisition, and this “cry for help” hypothesis is shown for plants upon encountering pathogens (Berendsen et al., 2012; Kwak et al., 2018; Yuan et al., 2018). It is interesting that our functional prediction of enriched bacterial taxa in the rhizoplane of clover and maize showed significant differences in terms of functional genes relevant for P metabolism (Figures 6A,B) and C cycling (Figures 7A,B) in maize and clover, respectively. Functional genes related to C cycling were significantly different between P_5_ and P_30_ in maize (Figures 6A,B), whilst differences in functional genes related to P metabolism were observed in clover (Figures 7A,B). Atmospheric N_2_-fixation by clover, which is an energy- and hence P-demanding process, is likely linked to that difference. N_2_-fixing legumes exhibited high root phosphatase activity especially at low soil P availability (Png et al., 2017). Maize is a C_4_ plants and under low P soil carbon availability may constrain the recruitment of soil microbiome. The importance of C metabolism in rhizosphere microbial assembly has been reported for sugarcane (Hamonts et al., 2018). Metagenomic analysis on cucumber and wheat identified a core set of functional genes, including functional gene profile of C and nitrate assimilation (Ofek et al., 2014). In addition, bacterial interactions may also affect microbial assembly in maize rhizosphere (Niu et al., 2017). How microbes interact to affect P acquisition by host plants needs to be further dissected.

The need to increase P fertilizer use efficiency may lead to the utilization of microorganisms to support P cycling in agroecosystems. A negative microbial legacy was only observed in monoculture maize but not in clover. Hierarchical habitat filtering of bacterial communities was observed in the rhizoplane of the two plant species, and rhizocompartment was the main filter determining diversity and structure of bacterial communities. Specific OTUs were consecutively enriched from bulk soil to rhizoplane, and these dominant OTUs in the rhizoplane collectively exhibited different functions in relation to P-metabolism in clover and C-metabolism by maize. Our results indicated that the microbial legacy effect by long-term P fertilization is overridden by host identity and rhizocompartment. These results highlight the importance of crop diversification for improving P efficiency. The close linkages between rhizoplane microbiome with host plant metabolism indicate that the functions of microbial communities should be integrated into P management to increase P use efficiency and sustainable food production.

## Data Availability Statement

The datasets generated or analyzed during the current study are available in the NCBI SRA repository under the BioProject ID: langming (accession number SRP139180).

## Author Contributions

JZ and ML conceived the study. ML and SB contributed to the data analysis of bioinformatics. All authors contributed to the data interpretation and manuscript preparation, critically reviewed and edited the manuscript, and approved it for publication.

## Conflict of Interest

The authors declare that the research was conducted in the absence of any commercial or financial relationships that could be construed as a potential conflict of interest.

## Footnotes

1 http://picrust.github.io/picrust/
