# Supplementary material for: Rhizoplane Bacteria and Plant Species Co-determine Phosphorus-Mediated Microbial Legacy Effect
Source: Front Microbiol. 2019 Dec 10;10:2856. doi: 10.3389/fmicb.2019.02856 (PMC6914688; doi:10.3389/fmicb.2019.02856)
Supplement: Supplementary file 1 [file Data_Sheet_1.docx]

***Supplementary Material***

**Rhizoplane bacteria and plant species co-determine phosphorus-mediated microbial legacy effect**

**Running title: Host overrides microbial P legacy**

Ming Lang^1, 2^, Shuikuan Bei^2^, Xia Li^2,3^, Thomas W. Kuyper^4^ and Junling Zhang^2*^

^1^College of Resources and Environment, Southwest University, Chongqing, China

^2^Centre for Resources, Environment and Food Security, College of Resources and Environmental Sciences, China Agricultural University; Key Laboratory of Plant-Soil Interactions, Ministry of Education, Beijing, China

^3^School of Life Science, Shanxi Datong University, Datong, China

^4^Wageningen University, Soil Biology Group, P.O. Box 47, 6700 AA, Wageningen, The Netherlands

***Correspondence:**

Junling Zhang

Junlingz@cau.edu.cn

**
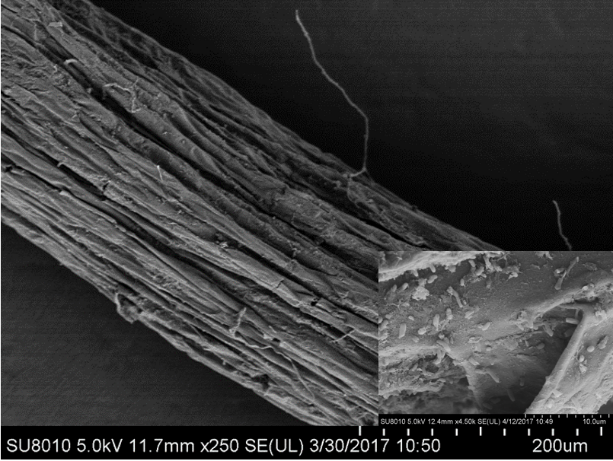

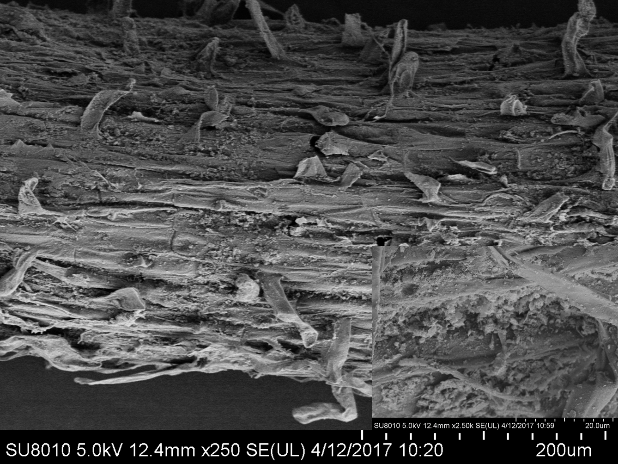
**

Figure S1: Representative pictures of clover roots subjected to (+) or not (-) to sonication using scanning electron microscopy (SEM).

Figure S2: Bacterial communities of unsterilized and sterilized bulk soil of maize and clover plants. Principal coordinate analysis (PCoAs) plots of the OTU-based Bray-Curtis distance. The variance explained by each PC axis is given in parentheses.


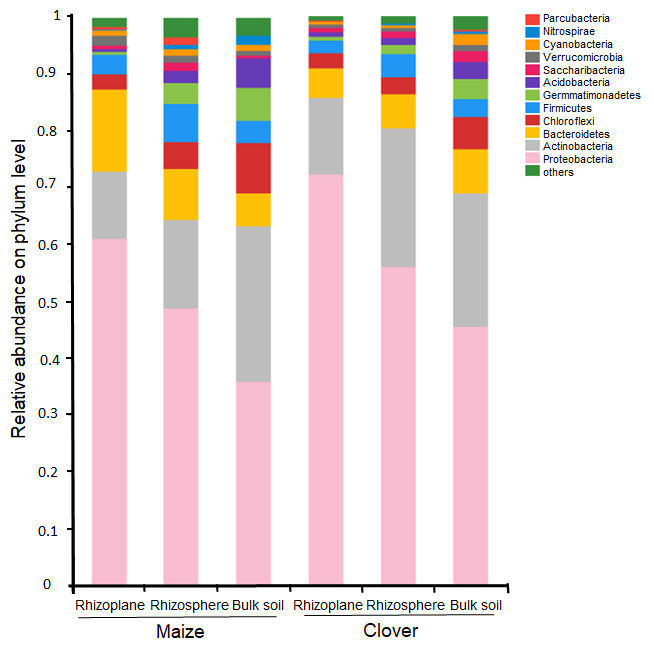


Figure S3: Mean relative abundance (%) of the dominant phyla in the rhizocompartments (rhizoplane, rhizosphere and bulk soil) of maize and clover. Values are indicated for phyla with > 1% average relative abundance.


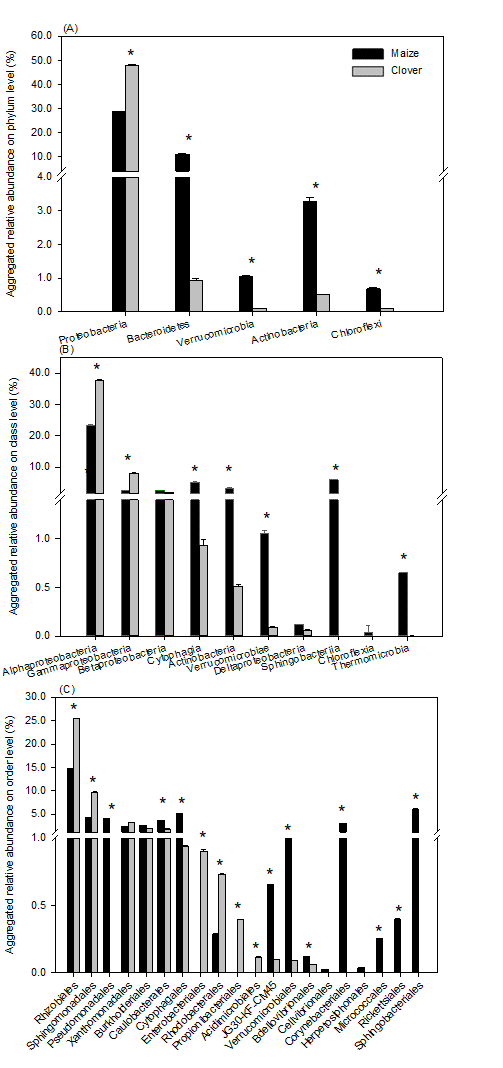


Figure S4: Bar chart of the consecutively enriched relative abundance aggregated to the (A) phylum (n = 48), (B) class (n = 48) and (C) order (n = 48) taxonomic level in the rhizoplane of maize and clover plants.

Figure S5: Networks in the rhizoplane, rhizosphere and bulk soil of clover and maize grown in soils amended with two substrate P levels (P5, P30: 5 or 30 mg P kg-1 soil). The networks were based on the co-occurrence network analysis from OTU profiles. Color dots represented different bacterial OUT taxa.

Table S1: Available P (Olsen-P), P concentration and content of shoots of clover and maize plants grown in soils amended with different P supply (P_5_, P _30_: 5 or 30 mg P kg^-1^ soil respectively), and inoculated with soils consistently unfertilized (I_0_), or fertilized with 33 (I_33_) or 131 (I_131_) kg P ha^-1^ year^-1^ for 9 years.

| Soil P | Inoculum | Inoculum type | Maize | | |  | Clover | | |
| --- | --- | --- | --- | --- | --- | --- | --- | --- | --- |
|  |  |  | Available P | Shoot P concentration | Shoot P content |  | Available P | Shoot P concentration | Shoot P content |
| mg kg^-1^ | kg P ha^-1^ |  | mg g^-1^ | mg g^-1^ | mg pot^-1^ |  | mg g^-1^ | mg g^-1^ | mg pot^-1^ |
| P_5_ | I_0_ | Sterilized | 4.72±0.14 a | 0.85±0.06 a | 0.75±0.19 a |  | 4.16±0.06 a | 0.81±0.01 a | 0.33±0.01 a |
| P_5_ | I_33_ | Sterilized | 4.69±0.45 a | 0.58±0.05 a | 1.03±0.36 a |  | 4.98±0.46 a | 1.15±0.26 a | 0.22±0.02 b |
| P_5_ | I_131_ | Sterilized | 4.97±0.10 a | 0.68±0.02 a | 0.62±0.01 a |  | 5.29±0.40 a | 0.82±0.01 a | 0.37±0.03 a |
|  |  |  | A | B | B |  | A | B | B |
| P_5_ | I_0_ | Inoculum | 4.54±0.18 b | 0.88±0.15 a | 0.77±0.06 b |  | 3.34±0.28 a | 1.09±0.10 ab | 1.45±0.44 a |
| P_5_ | I_33_ | Inoculum | 4.72±0.17 b | 1.07±0.17 a | 0.59±0.06 b |  | 3.63±0.15 a | 0.81±0.02 b | 1.80±0.27 a |
| P_5_ | I_131_ | Inoculum | 5.49±0.20 a | 0.83±0.05 a | 2.33±0.27 a |  | 3.62±0.18 a | 1.65±0.30 a | 1.61±0.08 a |
|  |  |  | A | A | A |  | B | A | A |
| P_30_ | I_0_ | Sterilized | 8.45±0.24 a | 1.17±0.06 a | 2.33±0.27 a |  | 8.21±0.55 a | 1.07±0.07 a | 1.73±0.10 a |
| P_30_ | I_33_ | Sterilized | 8.47±0.46 a | 1.32±0.25 a | 2.89±0.14 a |  | 8.45±0.14 a | 1.16±0.10 a | 1.53±0.24 a |
| P_30_ | I_131_ | Sterilized | 7.99±0.31 a | 1.11±0.07 a | 2.87±0.22 a |  | 9.05±0.22 a | 1.12±0.04 a | 1.67±0.09 a |
|  |  |  | A | B | A |  | A | A | B |
| P_30_ | I_0_ | Inoculum | 7.80±0.43 a | 1.79±0.09 a | 2.46±0.81 b |  | 7.07±0.57 a | 1.50±0.38 a | 3.57±0.19 a |
| P_30_ | I_33_ | Inoculum | 7.28±0.45 a | 1.53±0.21 a | 2.27±0.05 b |  | 7.28±0.10 a | 1.10±0.07 a | 2.78±0.41 a |
| P_30_ | I_131_ | Inoculum | 7.14±0.12 a | 1.51±0.12 a | 4.74±0.61 a |  | 7.99±0.42 a | 1.78±0.30 a | 3.32±0.52 a |
|  |  |  | B | A | A |  | A | A | A |

Data are mean ± SEM (n = 4). Significant differences among three inoculum treatments and P supply levels within each variable are indicated by dissimilar lowercase or uppercase letters, respectively (*P* < 0.05).

Table S2: Bacterial beta diversity of (A) maize and (B) clover as affected by rhizocompartment, P supply level and inoculum

(A)

|  | Df | Sum of Sqs | Mean Sqs | F. Model | R^2^ | *P* |
| --- | --- | --- | --- | --- | --- | --- |
| Rhizocompartment (R) | 2 | 6.6327 | 3.3163 | 35.311 | 0.44039 | < 0.001 |
| Substrate P level (P) | 1 | 0.3259 | 0.3259 | 3.47 | 0.02164 | 0.009 |
| Inoculum (I) | 2 | 0.4698 | 0.2349 | 2.501 | 0.03119 | 0.010 |
| R * P | 2 | 0.6059 | 0.303 | 3.226 | 0.04023 | 0.003 |
| R * I | 4 | 0.7523 | 0.1881 | 2.003 | 0.04995 | 0.012 |
| P * I | 2 | 0.5126 | 0.2563 | 2.729 | 0.03403 | 0.004 |
| R * P * I | 4 | 0.878 | 0.2195 | 2.337 | 0.0583 | 0.002 |
| Residuals | 54 | 4.8837 | 0.0939 |  | 0.32426 |  |
| Total | 71 | 15.061 |  |  | 1 |  |

(B)

|  | Df | Sum of Sqs | Mean Sqs | F. Model | R^2^ | *P* |
| --- | --- | --- | --- | --- | --- | --- |
| Rhizocompartment (R) | 2 | 2.4425 | 1.22125 | 17.7418 | 0.30571 | < 0.001 |
| Substrate P level (P) | 1 | 0.2186 | 0.21862 | 3.176 | 0.02736 | 0.007 |
| Inoculum (I) | 2 | 0.2076 | 0.1038 | 1.508 | 0.02598 | 0.077 |
| R * P | 2 | 0.4592 | 0.22958 | 3.3352 | 0.05747 | 0.001 |
| R * I | 4 | 0.3125 | 0.07811 | 1.1348 | 0.03911 | 0.285 |
| P * I | 2 | 0.2601 | 0.13006 | 1.8894 | 0.03256 | 0.022 |
| R * P * I | 4 | 0.3722 | 0.09304 | 1.3517 | 0.04658 | 0.078 |
| Residuals | 54 | 3.7171 | 0.06884 |  | 0.46523 |  |
| Total | 71 | 7.9897 |  |  | 1 |  |

Table S3: Permutational multivariate analyses on bacterial community structure in different rhizocompartments as affected by P supply level (P_5_, P_30_: 5 or 30 mg P kg^-1^ soil) based on Bray-Curtis distance

| Plant species | Rhizocompartment | Substrate P level | Df | Sums Of Sqs | Mean Sqs | F. Model | R^2^ | *P* |
| --- | --- | --- | --- | --- | --- | --- | --- | --- |
| Maize | Rhizoplane | P_5_ / P_30_ | 3 | 0.219 | 0.219 | 2.677 | 0.118 | 0.013 |
|  | Rhizosphere | P_5_ / P_30_ | 3 | 0.054 | 0.054 | 1.77 | 0.078 | 0.046 |
|  | Bulk soil | P_5_ / P_30_ | 3 | 0.216 | 0.216 | 4.778 | 0.185 | 0.015 |
| Clover | Rhizoplane | P_5_ / P_30_ | 3 | 0.137 | 0.137 | 3.768 | 0.146 | <0.001 |
|  | Rhizosphere | P_5_ / P_30_ | 3 | 0.231 | 0.231 | 6.454 | 0.227 | <0.001 |
|  | Bulk soil | P_5_ / P_30_ | 3 | 0.125 | 0.125 | 5.064 | 0.187 | <0.001 |

Table S4: Constrained principal coordinates analysis (CAPs) using the Bray-Curtis distances generated for each rhizocompartment of clover and maize plants.

| Plant species | Rhizocompartment | Factor | Df | Variance | F | *P* value | Percent of variance |
| --- | --- | --- | --- | --- | --- | --- | --- |
| Maize | Rhizoplane | P level | 1 | 0.283 | 2.179 | 0.009 | 8.96 |
|  |  | Inoculum | 2 | 0.536 | 2.065 | 0.003 | 16.99 |
|  | Rhizosphere | P level | 1 | 0.192 | 2.444 | < 0.001 | 9.4 |
|  |  | Inoculum | 2 | 0.28 | 1.779 | < 0.001 | 13.68 |
|  | Bulk soil | P level | 1 | 0.454 | 3.879 | 0.008 | 14.19 |
|  |  | Inoculum | 2 | 0.403 | 1.724 | 0.111 | 12.62 |
| Clover | Rhizoplane | P level | 1 | 0.323 | 4.511 | < 0.001 | 22.55 |
|  |  | Inoculum | 2 | 0.228 | 1.592 | 0.034 | 15.92 |
|  | Rhizosphere | P level | 1 | 0.422 | 6.613 | < 0.001 | 22.43 |
|  |  | Inoculum | 2 | 0.183 | 1.432 | 0.099 | 9.72 |
|  | Bulk soil | P level | 1 | 0.27 | 4.432 | 0.042 | 16.23 |
|  |  | Inoculum | 2 | 0.175 | 1.435 | < 0.001 | 7.32 |

Table S5: Permutational multivariate analysis of the effects of original and sterilized inoculum treatments on bacterial communities.

| Source of Variation | Bacterial community | | |
| --- | --- | --- | --- |
|  | F | R^2^ | *P* |
| Original and sterilized inoculum treatments (Maize) | 7.495 | 0.212 | 0.001 |
| Original and sterilized inoculum treatments (Clover) | 4.561 | 0.142 | 0.003 |

Table S6: Topological properties of the co-occurrence network of bacterial communities in rhizoplane, rhizosphere and bulk soil of clover and maize plants grown in soils amended with different substrate P supply levels (P_5_, P_30_; 5 or 30 mg P kg^-1^ soil).

| Plant species | Substrate P level | Rhizocompartments | Number of nodes | Number of edges | Modularity | Average path length | Average clustering  coefficient | Average  connectivity |
| --- | --- | --- | --- | --- | --- | --- | --- | --- |
| Maize | P_5_ | RP | 148 | 2653 | 2.01 | 1.83 | 0.52 | 1.82 |
| Maize | P_5_ | RS | 245 | 5890 | 1.08 | 1.91 | 0.50 | 1.91 |
| Maize | P_5_ | BS | 245 | 7570 | 4.88 | 1.34 | 0.68 | 1.34 |
| Maize | P_30_ | RP | 148 | 2664 | 1.46 | 1.83 | 0.46 | 1.82 |
| Maize | P_30_ | RS | 245 | 4702 | 1.11 | 2.01 | 0.52 | 1.98 |
| Maize | P_30_ | BS | 245 | 5922 | 1.39 | 1.98 | 0.57 | 2.00 |
| Clover | P_5_ | RP | 148 | 2490 | 3.73 | 1.97 | 0.56 | 1.96 |
| Clover | P_5_ | RS | 245 | 4671 | 3.40 | 2.10 | 0.50 | 2.09 |
| Clover | P_5_ | BS | 244 | 4817 | 2.70 | 2.05 | 0.49 | 2.04 |
| Clover | P_30_ | RP | 150 | 2491 | 2.22 | 1.89 | 0.53 | 1.89 |
| Clover | P_30_ | RS | 241 | 4810 | 2.85 | 2.02 | 0.52 | 1.96 |
| Clover | P_30_ | BS | 246 | 5836 | 3.14 | 1.97 | 0.46 | 2.02 |

Table S7: Classification of the top 10 bacteria based on the degree at each taxonomic level

| Plant species | Substract P level | Rhizocompartments | ID | Degree | Phylum | Class | Order | Family | Genus |
| --- | --- | --- | --- | --- | --- | --- | --- | --- | --- |
| Maize | P_5_ | Rhizoplane | OTU1034 | 59 | *Chlamydiae* | *Chlamydiae* | *Chlamydiales* | *Simkaniaceae* |  |
| Maize | P_5_ | Rhizoplane | OTU6373 | 57 | *Proteobacteria* | *Alphaproteobacteria* | *Rhizobiales* | *Methylobacteriaceae* |  |
| Maize | P_5_ | Rhizoplane | OTU654 | 56 | *Proteobacteria* | *Betaproteobacteria* | *Neisseriales* | *Neisseriaceae* |  |
| Maize | P_5_ | Rhizoplane | OTU3528 | 56 | *Chloroflexi* | *Chloroflexia* | *Chloroflexales* | *Roseiflexaceae* | *Roseiflexus* |
| Maize | P_5_ | Rhizoplane | OTU4086 | 56 | *Tectomicrobia* |  |  |  |  |
| Maize | P_5_ | Rhizoplane | OTU74 | 56 | *Saccharibacteria* |  |  |  |  |
| Maize | P_5_ | Rhizoplane | OTU4003 | 56 | *Proteobacteria* | *Deltaproteobacteria* | *Bdellovibrionales* | *Bdellovibrionaceae* | *Bdellovibrio* |
| Maize | P_5_ | Rhizoplane | OTU2282 | 55 | *Proteobacteria* | *Gammaproteobacteria* | *Xanthomonadales* | *Xanthomonadaceae* | *Lysobacter* |
| Maize | P_5_ | Rhizoplane | OTU4180 | 54 | *Gemmatimonadetes* | *Gemmatimonadetes* | *Gemmatimonadales* | *Gemmatimonadaceae* |  |
| Maize | P_5_ | Rhizoplane | OTU4048 | 53 | *Chlorobi* | *Chlorobia* | *Chlorobiales* | *OPB56* |  |
| Maize | P_5_ | Rhizosphere | OTU6724 | 210 | *Acidobacteria* | *Acidobacteria* |  |  |  |
| Maize | P_5_ | Rhizosphere | OTU2760 | 210 | *Proteobacteria* | *Gammaproteobacteria* | *Legionellales* | *Coxiellaceae* | Aquicella |
| Maize | P_5_ | Rhizosphere | OTU4556 | 210 | *Planctomycetes* | *Planctomycetacia* | *Planctomycetales* | *Planctomycetaceae* | Gemmata |
| Maize | P_5_ | Rhizosphere | OTU6043 | 210 | *Proteobacteria* | *Betaproteobacteria* | *Burkholderiales* | Oxalobacteraceae |  |
| Maize | P_5_ | Rhizosphere | OTU2814 | 209 | *Proteobacteria* | *Betaproteobacteria* | *Burkholderiales* | Comamonadaceae | Hydrogenophaga |
| Maize | P_5_ | Rhizosphere | OTU590 | 209 | *Acidobacteria* | *Acidobacteria* |  |  |  |
| Maize | P_5_ | Rhizosphere | OTU6093 | 209 | *Proteobacteria* | *Gammaproteobacteria* | Legionellales | Coxiellaceae | Aquicella |
| Maize | P_5_ | Rhizosphere | OTU6507 | 208 | *Chloroflexi* | *Chloroflexia* | Kallotenuales | Kallotenuaceae | Kallotenue |
| Maize | P_5_ | Rhizosphere | OTU381 | 208 | *Acidobacteria* | *Acidobacteria* |  |  |  |
| Maize | P_5_ | Rhizosphere | OTU6971 | 208 | *Proteobacteria* | *Gammaproteobacteria* | *Xanthomonadales* |  |  |
| Maize | P_5_ | Bulk | OTU5320 | 210 | *Proteobacteria* | *Alphaproteobacteria* | *Rhizobiales* | *Phyllobacteriaceae* |  |
| Maize | P_5_ | Bulk | OTU2367 | 210 | *Proteobacteria* | *Gammaproteobacteria* | *PYR10d3* |  |  |
| Maize | P_5_ | Bulk | OTU4869 | 210 | *Nitrospirae* | *Nitrospira* |  |  |  |
| Maize | P_5_ | Bulk | OTU3686 | 210 | *Acidobacteria* | *Acidobacteria* |  |  |  |
| Maize | P_5_ | Bulk | OTU1312 | 209 | *Chloroflexi* | *Anaerolineae* | *Anaerolineales* | *Anaerolineaceae* |  |
| Maize | P_5_ | Bulk | OTU6724 | 209 | *Acidobacteria* | *Acidobacteria* |  |  |  |
| Maize | P_5_ | Bulk | OTU2795 | 209 | *Chloroflexi* |  |  |  |  |
| Maize | P_5_ | Bulk | OTU361 | 208 | *Gemmatimonadetes* | *Gemmatimonadetes* | *Longimicrobiales* | *Longimicrobiaceae* |  |
| Maize | P_5_ | Bulk | OTU3872 | 208 | *Nitrospirae* | *Nitrospira* |  |  |  |
| Maize | P_5_ | Bulk | OTU6154 | 208 | *Proteobacteria* | *Betaproteobacteria* | *Burkholderiales* | Comamonadaceae | Aquabacterium |
| Maize | P_30_ | Rhizoplane | OTU4003 | 60 | *Proteobacteria* | *Deltaproteobacteria* | *Bdellovibrionales* | *Bdellovibrionaceae* | *Bdellovibrio* |
| Maize | P_30_ | Rhizoplane | OTU3528 | 59 | *Chloroflexi* | *Chloroflexia* | *Chloroflexales* | *Roseiflexaceae* | *Roseiflexus* |
| Maize | P_30_ | Rhizoplane | OTU1034 | 59 | *Chlamydiae* | *Chlamydiae* | *Chlamydiales* | *Simkaniaceae* |  |
| Maize | P_30_ | Rhizoplane | OTU2367 | 57 | *Proteobacteria* | *Gammaproteobacteria* | *PYR10d3* |  |  |
| Maize | P_30_ | Rhizoplane | OTU4180 | 56 | *Gemmatimonadetes* | *Gemmatimonadetes* | *Gemmatimonadales* | *Gemmatimonadaceae* |  |
| Maize | P_30_ | Rhizoplane | OTU4086 | 56 | *Tectomicrobia* |  |  |  |  |
| Maize | P_30_ | Rhizoplane | OTU2282 | 56 | *Proteobacteria* | *Gammaproteobacteria* | *Xanthomonadales* | *Xanthomonadaceae* |  |
| Maize | P_30_ | Rhizoplane | OTU74 | 55 | *Saccharibacteria* |  |  |  |  |
| Maize | P_30_ | Rhizoplane | OTU6373 | 54 | *Proteobacteria* | *Alphaproteobacteria* | *Rhizobiales* | *Methylobacteriaceae* |  |
| Maize | P_30_ | Rhizoplane | OTU2358 | 54 | *Actinobacteria* | *Actinobacteria* | *Acidimicrobiales* | *Acidimicrobiaceae* |  |
| Maize | P_30_ | Rhizosphere | OTU2216 | 92 | *Actinobacteria* | *Actinobacteria* | Gaiellales |  |  |
| Maize | P_30_ | Rhizosphere | OTU4100 | 84 | *Proteobacteria* | *Gammaproteobacteria* | Legionellales | Coxiellaceae | Aquicella |
| Maize | P_30_ | Rhizosphere | OTU6724 | 84 | *Acidobacteria* | *Acidobacteria* |  |  |  |
| Maize | P_30_ | Rhizosphere | OTU2272 | 81 | *Firmicutes* | *Bacilli* | Lactobacillales | Streptococcaceae |  |
| Maize | P_30_ | Rhizosphere | OTU6515 | 80 | *Acidobacteria* | *Acidobacteria* | *Solibacterales* | *Solibacteraceae__Subgroup_3_* |  |
| Maize | P_30_ | Rhizosphere | OTU3198 | 80 | *Proteobacteria* | *Deltaproteobacteria* | *Oligoflexales* | *0319-6G20* |  |
| Maize | P_30_ | Rhizosphere | OTU5319 | 80 | *Proteobacteria* | *Alphaproteobacteria* | *Rhodospirillales* | *I-10* |  |
| Maize | P_30_ | Rhizosphere | OTU6503 | 79 | *Acidobacteria* | *Acidobacteria* | *Subgroup_10* | *ABS-19* |  |
| Maize | P_30_ | Rhizosphere | OTU381 | 78 | *Acidobacteria* | *Acidobacteria* |  |  |  |
| Maize | P_30_ | Rhizosphere | OTU1307 | 78 | *Chloroflexi* | *Anaerolineae* | *Anaerolineales* | *Anaerolineaceae* |  |
| Maize | P_30_ | Bulk | OTU1191 | 117 | *Parcubacteria* |  |  |  |  |
| Maize | P_30_ | Bulk | OTU2740 | 111 | *Actinobacteria* | *Actinobacteria* | *Pseudonocardiales* | *Pseudonocardiaceae* | *Saccharopolyspora* |
| Maize | P_30_ | Bulk | OTU6934 | 111 | *Verrucomicrobia* | *Verrucomicrobiae* | *Verrucomicrobiales* | Verrucomicrobiaceae |  |
| Maize | P_30_ | Bulk | OTU5404 | 111 | *Bacteroidetes* | *Sphingobacteriia* | *Sphingobacteriales* | *Chitinophagaceae* |  |
| Maize | P_30_ | Bulk | OTU4456 | 109 | *Chloroflexi* | *Anaerolineae* | *Anaerolineales* | *Anaerolineaceae* |  |
| Maize | P_30_ | Bulk | OTU654 | 105 | *Proteobacteria* | *Betaproteobacteria* | *Neisseriales* | *Neisseriaceae* |  |
| Maize | P_30_ | Bulk | OTU6724 | 105 | *Acidobacteria* | *Acidobacteria* |  |  |  |
| Maize | P_30_ | Bulk | OTU3720 | 105 | *Saccharibacteria* |  |  |  |  |
| Maize | P_30_ | Bulk | OTU1312 | 102 | *Chloroflexi* | *Anaerolineae* | *Anaerolineales* | *Anaerolineaceae* |  |
| Maize | P_30_ | Bulk | OTU3669 | 102 | *Proteobacteria* | *Deltaproteobacteria* | *Desulfurellales* | *Desulfurellaceae* | *H16* |
| Clover | P_5_ | Rhizoplane | OTU5828 | 75 | *Bacteroidetes* | *Sphingobacteriia* | *Sphingobacteriales* | *Chitinophagaceae* |  |
| Clover | P_5_ | Rhizoplane | OTU4122 | 73 | *Chloroflexi* | *TK10* |  |  |  |
| Clover | P_5_ | Rhizoplane | OTU6019 | 69 | *Proteobacteria* | *Betaproteobacteria* | *TRA3-20* |  |  |
| Clover | P_5_ | Rhizoplane | OTU4249 | 67 | *Elusimicrobia* | *Elusimicrobia* | *MVP-88* |  |  |
| Clover | P_5_ | Rhizoplane | OTU3530 | 64 | *Proteobacteria* | *Deltaproteobacteria* | *Myxococcales* | *Sandaracinaceae* |  |
| Clover | P_5_ | Rhizoplane | OTU6697 | 64 | *Gemmatimonadetes* | *Gemmatimonadetes* | *Gemmatimonadales* | *Gemmatimonadaceae* | *Gemmatirosa* |
| Clover | P_5_ | Rhizoplane | OTU6419 | 63 | *Proteobacteria* | *Gammaproteobacteria* | *Legionellales* | *Coxiellaceae* |  |
| Clover | P_5_ | Rhizoplane | OTU6724 | 63 | *Acidobacteria* | *Acidobacteria* |  |  |  |
| Clover | P_5_ | Rhizoplane | OTU2378 | 62 | *Actinobacteria* | *Actinobacteria* | *Acidimicrobiales* |  |  |
| Clover | P_5_ | Rhizoplane | OTU608 | 61 | *Proteobacteria* | *Gammaproteobacteria* | *HTA4* |  |  |
| Clover | P_5_ | Rhizosphere | OTU3669 | 98 | *Proteobacteria* | *Deltaproteobacteria* | *Desulfurellales* | *Desulfurellaceae* | *H16* |
| Clover | P_5_ | Rhizosphere | OTU6506 | 97 | *Proteobacteria* | *Alphaproteobacteria* | *Sphingomonadales* | *Sphingomonadaceae* | *Sphingomonas* |
| Clover | P_5_ | Rhizosphere | OTU2054 | 96 | *Actinobacteria* | *Actinobacteria* | *Micromonosporales* | *Micromonosporaceae* | *Catellatospora* |
| Clover | P_5_ | Rhizosphere | OTU5577 | 95 | *Gemmatimonadetes* | *Gemmatimonadetes* |  |  |  |
| Clover | P_5_ | Rhizosphere | OTU4408 | 90 | *Proteobacteria* | *Alphaproteobacteria* | *Rhizobiales* | *Rhizobiales_Incertae_Sedis* | *Rhizomicrobium* |
| Clover | P_5_ | Rhizosphere | OTU1476 | 90 | *Acidobacteria* | *Acidobacteria* | *Subgroup_10* | ABS-19 |  |
| Clover | P_5_ | Rhizosphere | OTU5828 | 89 | *Bacteroidetes* | *Sphingobacteriia* | *Sphingobacteriales* | Chitinophagaceae |  |
| Clover | P_5_ | Rhizosphere | OTU1342 | 85 | *Proteobacteria* | *Deltaproteobacteria* |  |  |  |
| Clover | P_5_ | Rhizosphere | OTU5829 | 82 | *Actinobacteria* | *Actinobacteria* | *Acidimicrobiales* |  |  |
| Clover | P_5_ | Rhizosphere | OTU6570 | 82 | *Chloroflexi* | *TK10* |  |  |  |
| Clover | P_5_ | Bulk | OTU6022 | 107 | *Proteobacteria* | *Gammaproteobacteria* | *Xanthomonadales* | *Xanthomonadaceae* | *Lysobacter* |
| Clover | P_5_ | Bulk | OTU6433 | 96 | *Chloroflexi* | *Ardenticatenia* |  |  |  |
| Clover | P_5_ | Bulk | OTU1651 | 94 | *Actinobacteria* | *Actinobacteria* | *Acidimicrobiales* |  |  |
| Clover | P_5_ | Bulk | OTU2814 | 93 | *Proteobacteria* | *Betaproteobacteria* | *Burkholderiales* | *Comamonadaceae* | *Hydrogenophaga* |
| Clover | P_5_ | Bulk | OTU361 | 91 | *Gemmatimonadetes* | *Gemmatimonadetes* | *Longimicrobiales* | *Longimicrobiaceae* |  |
| Clover | P_5_ | Bulk | OTU6036 | 88 | *Proteobacteria* | *Betaproteobacteria* | *Burkholderiales* | *Comamonadaceae* |  |
| Clover | P_5_ | Bulk | OTU2432 | 86 | *Firmicutes* | *Bacilli* | *Bacillales* | Thermoactinomycetaceae | Laceyella |
| Clover | P_5_ | Bulk | OTU6336 | 85 | *Proteobacteria* | *Deltaproteobacteria* | *Bdellovibrionales* | *Bacteriovoracaceae* | *Peredibacter* |
| Clover | P_5_ | Bulk | OTU2901 | 85 | *Proteobacteria* | *Betaproteobacteria* | Nitrosomonadales | Nitrosomonadaceae |  |
| Clover | P_5_ | Bulk | OTU6724 | 84 | *Acidobacteria* | *Acidobacteria* |  |  |  |
| Clover | P_30_ | Rhizoplane | OTU4512 | 61 | *Proteobacteria* | *Deltaproteobacteria* | *Myxococcales* | *Haliangiaceae* | *Haliangium* |
| Clover | P_30_ | Rhizoplane | OTU6033 | 59 | *Proteobacteria* | *Gammaproteobacteria* | *Legionellales* | *Coxiellaceae* | *Aquicella* |
| Clover | P_30_ | Rhizoplane | OTU416 | 58 | *Proteobacteria* | *Gammaproteobacteria* | *Legionellales* | *Coxiellaceae* | *Aquicella* |
| Clover | P_30_ | Rhizoplane | OTU4180 | 57 | *Gemmatimonadetes* | *Gemmatimonadetes* | *Gemmatimonadales* | *Gemmatimonadaceae* |  |
| Clover | P_30_ | Rhizoplane | OTU6051 | 56 | *Proteobacteria* | *Gammaproteobacteria* | *Legionellales* | *Coxiellaceae* |  |
| Clover | P_30_ | Rhizoplane | OTU5828 | 54 | *Bacteroidetes* | *Sphingobacteriia* | *Sphingobacteriales* | *Chitinophagaceae* |  |
| Clover | P_30_ | Rhizoplane | OTU4654 | 54 | *Bacteroidetes* | *Sphingobacteriia* | *Sphingobacteriales* |  |  |
| Clover | P_30_ | Rhizoplane | OTU6154 | 53 | *Proteobacteria* | *Betaproteobacteria* | *Burkholderiales* | *Comamonadaceae* | *Aquabacterium* |
| Clover | P_30_ | Rhizoplane | OTU6515 | 53 | *Acidobacteria* | *Acidobacteria* | *Solibacterales* | *Solibacteraceae__Subgroup_3_* | *Bryobacter* |
| Clover | P_30_ | Rhizoplane | OTU2374 | 53 | *Chloroflexi* | *Anaerolineae* | *Anaerolineales* | *Anaerolineaceae* |  |
| Clover | P_30_ | Rhizosphere | OTU6056 | 94 | *Actinobacteria* | *Actinobacteria* | *Solirubrobacterales* | *Elev-16S-1332* |  |
| Clover | P_30_ | Rhizosphere | OTU3070 | 94 | *Actinobacteria* | *Actinobacteria* | *Propionibacteriales* | *Nocardioidaceae* |  |
| Clover | P_30_ | Rhizosphere | OTU5138 | 89 | *Proteobacteria* | *Deltaproteobacteria* | *Myxococcales* | *Sandaracinaceae* |  |
| Clover | P_30_ | Rhizosphere | OTU6570 | 86 | *Chloroflexi* | *TK10* |  |  |  |
| Clover | P_30_ | Rhizosphere | OTU1331 | 85 | *Proteobacteria* | *Gammaproteobacteria* | *Xanthomonadales* | *Xanthomonadaceae* |  |
| Clover | P_30_ | Rhizosphere | OTU6022 | 84 | *Proteobacteria* | *Gammaproteobacteria* | *Xanthomonadales* | *Xanthomonadaceae* | *Lysobacter* |
| Clover | P_30_ | Rhizosphere | OTU1342 | 84 | *Proteobacteria* | *Deltaproteobacteria* | *NB1-j* |  |  |
| Clover | P_30_ | Rhizosphere | OTU6936 | 83 | *Firmicutes* | *Bacilli* | *Bacillales* | *Paenibacillaceae* | Paenibacillus |
| Clover | P_30_ | Rhizosphere | OTU1990 | 83 | *Chloroflexi* | *Chloroflexia* | *Chloroflexales* | *Roseiflexaceae* | Roseiflexus |
| Clover | P_30_ | Rhizosphere | OTU2814 | 82 | *Proteobacteria* | *Betaproteobacteria* | *Burkholderiales* | Comamonadaceae | Hydrogenophaga |
| Clover | P_30_ | Bulk | OTU4304 | 103 | *Gemmatimonadetes* | *Gemmatimonadetes* | *Longimicrobiales* | *Longimicrobiaceae* |  |
| Clover | P_30_ | Bulk | OTU5331 | 100 | *Acidobacteria* | *Acidobacteria* |  |  |  |
| Clover | P_30_ | Bulk | OTU2729 | 99 | *Firmicutes* | *Clostridia* | *Halanaerobiales* | *ODP1230B8.23* |  |
| Clover | P_30_ | Bulk | OTU4455 | 99 | *Chloroflexi* | *Chloroflexia* | *Kallotenuales* | *AKIW781* |  |
| Clover | P_30_ | Bulk | OTU1679 | 99 | *Proteobacteria* | *Alphaproteobacteria* | *Rhizobiales* |  |  |
| Clover | P_30_ | Bulk | OTU2723 | 98 | *Proteobacteria* | *Gammaproteobacteria* | Xanthomonadales |  |  |
| Clover | P_30_ | Bulk | OTU1342 | 98 | *Proteobacteria* | *Deltaproteobacteria* | *NB1-j* |  |  |
| Clover | P_30_ | Bulk | OTU6033 | 98 | *Proteobacteria* | *Gammaproteobacteria* | *Legionellales* | Coxiellaceae | Aquicella |
| Clover | P_30_ | Bulk | OTU6503 | 98 | *Acidobacteria* | *Acidobacteria* | *Subgroup_10* | ABS-19 |  |
| Clover | P_30_ | Bulk | OTU6936 | 98 | *Firmicutes* | *Bacilli* | *Bacillales* | *Paenibacillaceae* | *Paenibacillus* |
